# Histone methyltransferase SETDB1 regulates liver cancer cell growth through methylation of p53

**DOI:** 10.1038/ncomms9651

**Published:** 2015-10-16

**Authors:** Qi Fei, Ke Shang, Jianhua Zhang, Shannon Chuai, Desheng Kong, Tianlun Zhou, Shijun Fu, Ying Liang, Chong Li, Zhi Chen, Yuan Zhao, Zhengtian Yu, Zheng Huang, Min Hu, Haiyan Ying, Zhui Chen, Yun Zhang, Feng Xing, Jidong Zhu, Haiyan Xu, Kehao Zhao, Chris Lu, Peter Atadja, Zhi-Xiong Xiao, En Li, Jianyong Shou

**Affiliations:** 1 Novartis Institutes for BioMedical Research, Shanghai 201203, China; 2 College of Life Sciences, Sichuan University, Chengdu 610064, China

## Abstract

SETDB1 is a histone H3K9 methyltransferase that has a critical role in early development. It is located within a melanoma susceptibility locus and facilitates melanoma formation. However, the mechanism by which SETDB1 regulates tumorigenesis remains unknown. Here we report the molecular interplay between SETDB1 and the well-known hotspot gain-of-function (GOF) *TP53* R249S mutation. We show that in hepatocellular carcinoma (HCC) *SETDB1* is overexpressed with moderate copy number gain, and GOF *TP53* mutations including R249S associate with this overexpression. Inactivation of SETDB1 in HCC cell lines bearing the R249S mutation suppresses cell growth. The *TP53* mutation status renders cancer cells dependent on SETDB1. Moreover, SETDB1 forms a complex with p53 and catalyses p53K370 di-methylation. SETDB1 attenuation reduces the p53K370me2 level, which subsequently leads to increased recognition and degradation of p53 by MDM2. Together, we provide both genetic and biochemical evidence for a mechanism by which SETDB1 regulates cancer cell growth via methylation of p53.

## Abstract (web summary)

SETDB1 is a histone methyltransferase and a role for the protein has been proposed in cancer. Here, the authors show that SETDB1 contributes to hepatocellular cancer by preferably forming a complex with mutant p53, resulting in di-methylation of a critical lysine residue and stabilization of the protein.

## Graphical abstract

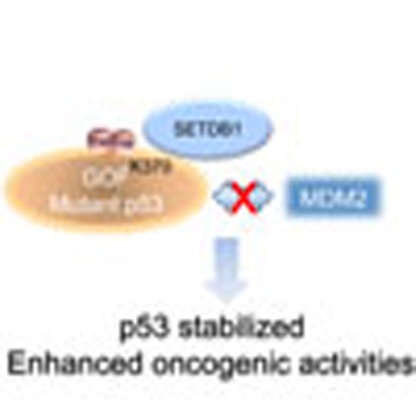

Hepatocellular carcinoma (HCC), the fifth most common cancer worldwide, is one of the most prevalent malignancies in Asian populations. Similar to other cancers, HCC is a heterogeneous disease driven by progressive genetic aberrations including silencing of tumour suppressor genes, oncogene activation and chromosomal anomalies. Epigenetic mechanisms often cooperate with genetic ones in the alteration of chromatin status that leads to the development of malignancy. *TP53*, which encodes the human tumour suppressor p53, is among the most frequently mutated genes in HCC. p53 functions as a transcription factor that regulates a large number of genes in response to a variety of cellular stress stimuli. Reportedly, *TP53* is mutated in ∼50% of human tumours including HCC^1^,^2^,^3^,^4^,^5^. The majority *TP53* monoallelic missense or nonsense mutations in the DNA-binding domain abrogate p53 DNA-binding specificity and lead to a loss of its tumour-suppressive nature. The mutated alleles can also act as a dominant-negative form to suppress the wild-type allele functions. Moreover, certain mutations may acquire oncogenic properties that are independent of the wild-type p53, which is known as the gain-of-function (GOF) *TP53* mutation^1^,^6^. GOF *TP53* mutations contribute to genomic instability, inactivation of P63 and P73, aberrant gene transcription, anti-apoptosis activity and enhanced tumour cell invasion and migration.

Normally, the wild-type p53 protein is kept at a very low level in cells. In response to cellular stress, such as DNA damage or hypoxia, it is rapidly stabilized and activated. However, the GOF p53 protein is often constitutively stable in tumour cells, and the accumulation of the mutant p53 is thought to be required for its oncogenic activities. The p53 activity is regulated through various post-translational modifications, including phosphorylation, acetylation, ubiquitination and methylation^7^,^8^,^9^. Recently, several histone methyltransferases (HMTs) and demethylase (HDMs) such as KMT7, KMT3c, KMT5A, EHMT2 and KDM1 are found to modulate the methylation status of p53 at distinct sites^10^,^11^,^12^,^13^,^14^,^15^. However, the methylase(s) responsible for the previously observed K370 di-methylation remained unidentified.

SETDB1 is an H3K9 methyltransferase that methylates histone H3 on lysine 9, up to tri-methylation (H3K9me3)^16^. It is recruited to the chromatin by the methyl-CpG-binding protein MBD1 (ref. 17) and silence genes including tumour suppressor genes, such as *RASSF1A* and *P53BP2*. In previous studies we have shown that *Setdb*1 is critical for embryonic development^18^. It is also involved in the counteraction between the Notch and Wnt/β-catenin pathways in colon cancer^19^,^20^. *SETDB1* is amplified in many tumour types, such as lung cancer^21^, sits within a melanoma susceptibility locus^22^ and facilitates melanoma formation in zebrafish^23^.

In the present study, we report that SETDB1 is overexpressed in HCC and that SETDB1 overexpression associates with p53 mutations. Moreover, GOF but not wild-type p53 status renders cells dependent on SETDB1. SETDB1 executes its role on cancer cell growth through di-methylating p53 at K370.

## Results

### *SETDB1* is overexpressed in liver cancer

It is reported that *SETDB1* is amplified in melanoma as well as in other cancer types including liver cancer^23^. Using the GISTIC (Genomic Identification of Significant Targets in Cancer) algorithm^24^, we analysed DNA copy number alternation in human cancer cell lines and primary tumour tissues. We confirmed that *SETDB1* was amplified in various tumour types including liver cancer as well as in many cancer cell lines similar to previous reports^23^. For example, in one study with 103 HCCs with hepatitis C virus infection (GSE9845), *SEDTB1* was adjacent (0.08 MB away) to the second most significant GISTIC amplification peak (*q* value=3e−25).

**Figure 1:**
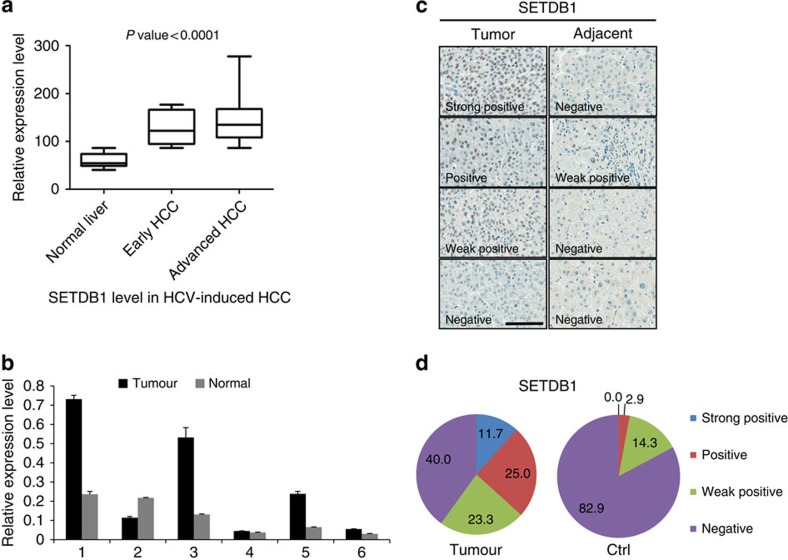
SETDB1 is overexpressed in liver cancers. (**a**) Publically available clinical cancer gene expression data (GSE6764) were downloaded from GEO to assess *SETDB1* expression in HCC tumour samples. The expression of *SETDB1* was extracted and box-plotted using Prism. (**b**) Total RNA was isolated from six pairs of human primary HCC tissues. The expression of *SETDB1* in these samples was analysed using RT–qPCR. Data are presented as mean±s.d. (**c**) Representative immunohistochemical staining of the SETDB1 protein using HCC TMA. The sections were counterstained with haematoxylin and eosin (H&E) staining. Scale bar, 100 μM. (**d**) Percentages of strong positive, positive, weak positive and negative samples for SETDB1 in tumours (*n*=59) and normal adjacent controls (*n*=35).

We next asked whether *SETDB1* is overexpressed in liver cancer. We surveyed publicly available gene expression data and found that *SETDB1* is highly expressed in various tumour types including breast, renal cell cancer (RCC) and liver cancers. In an Hepatitis C Virus (HCV)-induced HCC study (GSE6764), gene expression was measured at various stages of tumorigenesis. We found that the expression of *SETDB1* correlated well with the grade of tumorigenesis with later-stage cancer expressing higher level of *SETDB1* (Fig. 1a). The average expression level of *SETDB1* in late-stage liver cancer is significantly higher than that of the normal control (*t*-test, *P*<0.001). To corroborate these results, we assayed the expression of SETDB1 in six independent pairs of liver cancer samples with adjacent normal tissue as the control using reverse transcriptase quantitative PCR (RT–qPCR). In four out of the six pairs, SETDB1 expression is higher in tumour than in control (Fig. 1b).

To assess the protein levels of SETDB1 in liver cancer samples, we performed immunocytochemistry using tumour tissue microarray (TMA) with an antibody against SETDB1. The results are shown in Fig. 1c,d. We found that SETDB1 was highly expressed in tumour tissues relative to the adjacent normal controls. Sixty per cent (35 out of 59) tumours were positive for SETDB1, while only seventeen per cent (6 out of 35) were positive for the adjacent normal controls. From these experiments, we conclude that SETDB1 is overexpressed in a subset of liver cancer patients.

### *SETDB1* copy number gain associates with *TP53* mutations in HCC

**Figure 2:**
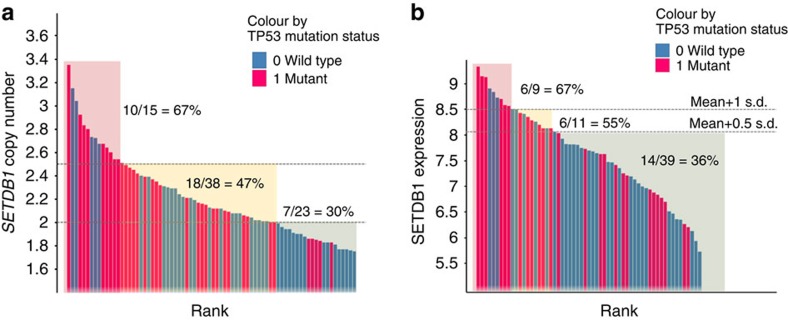
Association of TP53 mutations with SETDB1 copy number gain or overexpression in HCC tumours. Waterfall plots were generated to illustrate the association of *TP53* mutations with *SETDB1* copy number gain (**a**) or overexpression (**b**). Eighty-four HCC tumours were either ranked by *SETDB1* copy number or expression level. Each bar represents one tumour sample, which is coloured by the *TP53* mutation status. Tumours with *TP53* mutation are marked as red.

To explore genetic aberrations associated with *SETDB1* amplification and/or overexpression, we profiled 84 Asian HCC primary tumour tissue samples with exome sequencing, Human SNP array 6.0 and RNA expression microarrays. We found that *TP53* mutations were associated with *SETDB1* copy number gain (copy number >2.5) or overexpression (summarized in Table 1). We observed a statistically significantly increased proportion of *TP53* mutation among the HCC tumour samples carrying *SETDB1* copy number gain or overexpression (Fisher's exact test *P*=0.03; odds ratio=3.2, Fig. 2a,b). We also observed a trend of *TP53* mutation enrichment in gastric cancer with *SETDB1* copy number gain/overexpression, albeit not statistically significant. Among the 12 cases of *TP53* mutations found in liver cancer patients with *SETDB1* copy number gain/overexpression, four carried the hotspot R249S mutation.

R249S is a well-characterized GOF *TP53* hotspot mutation^5^,^6^,^25^. It is reported that the R249S mutation can increase cell migration and cell proliferation in lung cancer cells^26^,^27^. To evaluate the frequency of R249S mutation in Asian liver cancer patients, we genotyped an independent Chinese liver cancer cohort using restriction fragment length polymorphism analysis. In one group of DNA isolated from paraffin sections, we found that 91% (91 out of 100) of the tumour samples carried the R249S mutation (Supplementary Fig. 1a), consistent with previous reports^3^,^4^,^5^. The well-known P72R polymorphism was used as a control in the same set of DNA, and its pattern was apparently different from that of R249S, suggesting a low likelihood of DNA cross-contamination (Supplementary Fig. 1b). In another independent collection of 20 pairs of tumour and adjacent normal control samples, we found that 15% (3 out of 20) of the tumours carried the R249S mutation but none of the adjacent normal controls does.

### SETDB1 regulates the growth of HCC cell lines bearing R249S

**Figure 3:**
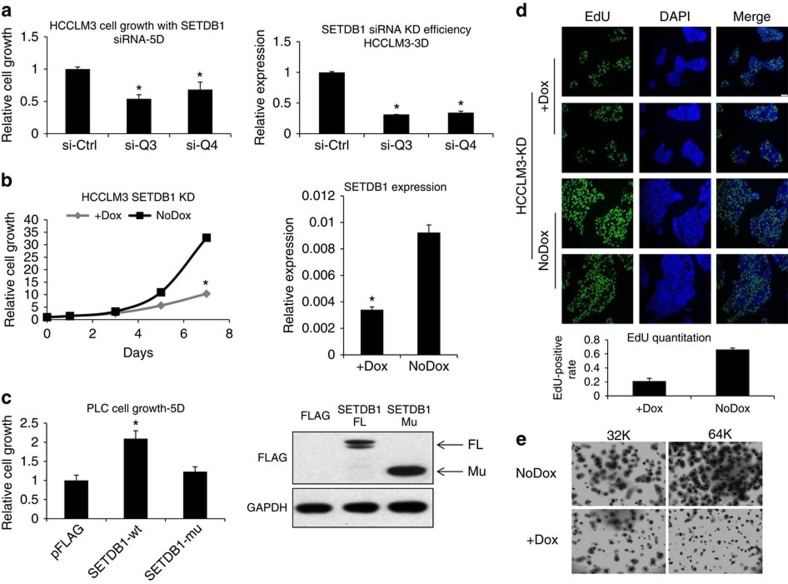
Inhibition of SETDB1 suppresses HCCLM3 cell growth *in vitro*. (**a**) HCCLM3 cells were transfected with two independent siRNAs against SETDB1. Cells were grown for 5 days and cell proliferation was measured with CellTiter-Glo (*n*=6). The knockdown efficiency of these two siRNAs was confirmed by using RT–qPCR (*n*=3). Data are presented as mean±s.d. *P<0.001, *t*-test. (**b**) Cells were infected with inducible lentiviral-based shRNA against SETDB1. Cells were treated with 1 μg ml^−1^ doxycycline to induce SETDB1 knockdown and cell growth was measured on days 3, 5 and 7, respectively. Data are presented as mean±s.d. *P<0.001, *t*-test, *n*=6. Target knockdown was confirmed using RT–qPCR. (**c**) PLC cells were transfected with FLAG-tagged SETDB1 overexpression construct or pcDNA3 vector control or a set domain-deleted SETDB1 mutant. Cell growth was measured 5 days after transfection. Data are presented as mean±s.d. *P<0.05, *t*-test, *n*=6. Expression level of the wild-type or mutant SETDB1 was confirmed with western blot analysis using the antibody against FLAG. FL, full length; Mu, mutated SETDB1. (**d**) HCCLM cells were treated with 1 μg ml^−1^ doxycycline for 3 days, and 10 μM EdU was added to the culture for the last 6 h of culture. EdU incorporation was assessed by immunofluorescence using the antibody against EdU. Two representative images of the control and SETDB1 knockdown cells are shown here. Inhibition of SETDB1 significantly reduced the number of EdU-positive cells. The relative ratios of the EdU-positive cells are also plotted. SETDB1 knockdown significantly reduced the number of EdU-positive cells. (**e**) HCCLM3 cells were grown in three-dimension in soft agar at the density of 32 or 64K cells ml^−1^. Cells were treated with doxycycline for 3 or 4 days, and the cultures were stained for *p*-iodonitrotetrazolium violet overnight at 37 °C.

To explore the potential roles of SETDB1 in HCC, we profiled *SETDB1* expression and examined the *TP53* mutation status in a panel of HCC cell lines (Supplementary Fig. 2). Three cell lines (HCCLM3, PLC/PRF and JHH4) were found with endogenous R249S mutation. Among them, HCCLM3 also has a *SETDB1* copy number gain with relatively high SETDB1 expression. Inhibition of SETDB1 using short interfering RNAs (siRNAs) suppressed cell proliferation of HCCLM3 cells (Fig. 3a). To further confirm these results, we generated stable cancer cell lines expressing inducible short hairpin RNAs (shRNAs) against SETDB1. Similar growth inhibitory effects were observed by induction of SETDB1 shRNAs (Fig. 3b). The two cell lines with R249S mutation but not SETDB1 copy number gain (JHH4 and PLC/PRF) were moderately inhibited when SETDB1 was attenuated (Supplementary Fig. 3).

We then asked whether SETDB1 overexpression drives cancer cell growth. Since gene transfection efficiency is low in HCCLM3 cells, we addressed this in the PLC/PRF cell line. The cell proliferation was stimulated by exogenous expression of wild-type SETDB1, but not set domain-truncated SETDB1 (Fig. 3c). Western blot analysis confirmed that the protein expression level of the mutant SETDB1 is not less than that of the wild type. Our findings suggest that SETDB1 promotes liver cancer cell growth, which depends on its methylation enzymatic activity.

To address whether SETDB1 inhibition affects DNA synthesis, we treated HCCLM3 cells with a pulse of 5-ethynyl-2′-deoxyuridine (EdU) on SETDB1 knockdown. We found that the percentage of EdU-positive cells was significantly reduced on SETDB1 knockdown (Fig. 3d), suggesting that SETDB1 inhibition reduced the number of cells in the S phase. In addition, inhibition of SETDB1 using inducible shRNA significantly inhibited anchorage-independent growth of HCCLM3 cells in a three-dimensional soft agar assay (Fig. 3e).

### *TP53* mutation confers cancer cell sensitivity to SETDB1

**Figure 4:**
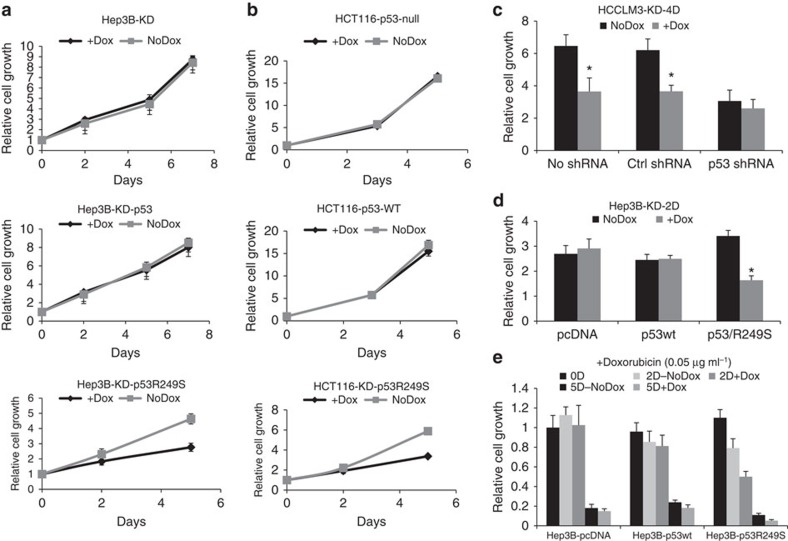
R249S mutation renders cancer cell sensitive to SETDB1 knockdown. (**a**) Parental or wild-type p53 or p53R249S-expressing Hep3B cells were infected with an inducible lentiviral-based shRNA against SETDB1. Cells were treated with 1 μg ml^−1^ doxycycline to induce SETDB1 knockdown, and cell growth was measured on days 2, 5 and 7. Data are presented as mean±s.d. *n*=6. (**b**) Assessing growth phenotypes on SETDB1 knockdown in isogenic pair of somatic p53 knockout HCT116 cells, or R249S restored HCT116 cells. Cells were infected with SETDB1 shRNA, and growth was measured on days 3 and 5, the same as above. Data are presented as mean±s.d. *n*=6. (**c**) Growth inhibition on SETDB1 knockdown was measured in HCCLM3 with stably infected SETDB1-inducible shRNA. The cells were treated with a control scramble shRNA or shRNA against p53. Cell growth assessment was performed the same as above. Data are presented as mean±s.d; *P<0.05, *t*-test, *n*=6. (**d**) Hep3B cells with stably infected SETDB1 shRNA were transfected with vector control, wild-type p53 or p53R249S mutants. Growth inhibition on SETDB1 knockdown was measured 2 days after transfection. Data are presented as mean±s.d.; *P<0.05, *t*-test, *n*=6. (**e**) Cell growth analysis was performed the same as above, except that the cells were treated with 0.05 μg ml^−1^ Doxorubicin for 2 or 5 days. Data are presented as mean±s.d; *P<0.05, *t*-test, *n*=6.

To address whether the inhibition of cell growth by SETDB1 depends on the p53 status, we examined SETDB1 knockdown in p53 null Hep3B cells, which also contain high *SETDB1* expression. Interestingly, Hep3B cells were insensitive to SETDB1 knockdown (Fig. 4a), suggesting that the *SETDB1* expression level *per se* does not determine the cancer cell dependence. Knockdown of SETDB1 in wild-type p53-restored Hep3B did not affect cell growth either (Fig. 4a). We also performed SETDB1 knockdown experiments using the isogenic pair of HCT116 cells in which p53 is somatically deleted or intact. Knockdown of SETDB1 showed no growth inhibition in either wild-type or p53-deleted cells (Fig. 4b). Together, the data suggest that wild-type p53 does not dictate SETDB1 dependence in cancer cells.

Since R249S is a GOF mutation of *TP53*, we then asked whether the R249S mutation would confer cell sensitivity to SETDB1 knockdown. We first attenuated p53 in HCCLM3 using shRNA against p53. We found that SETDB1 knockdown no longer inhibited cell growth in case of p53 abolishing (Fig. 4c). The knockdown efficiency of p53 shRNA was confirmed using PCR analysis (Supplementary Fig. 4). We next expressed p53R249S in p53 null Hep3B cells. We found that R249S-expressing cells became sensitive to SETDB1 knockdown (Fig. 4d). We next treated the wild-type or R249S p53-restored Hep3B cells with a DNA damage inducer, Doxorubicin. Under such conditions, cell growth was inhibited on SETDB1 inhibition only in the cells expressing R249S but not in wild-type p53 (Fig. 4e). p53R249S can render both Hep3B and HCT116 cells sensitive to SETDB1 knockdown (lowest panel, Fig. 4a,b). Together, our data strongly suggest that R249S, not the wild-type p53, determines the cell growth sensitivity to SETDB1 knockdown.

### SETDB1 complex di-methylates p53 at the 370 lysine residue

To understand the interplay between p53 and SETDB1, we asked whether SEDTB1 could form a complex with p53 protein in cells. We therefore treated HCCLM3 cells with Doxorubicin, followed by co-immunoprecipitation experiment and found that SETDB1 was indeed associated with p53 (data not shown). We also transfected HCT116 p53 null cells with Flag-tagged SETDB1 and wild-type or R249S-mutated p53. Immunoprecipitation results indicated that both wild-type and mutant p53 can form complex with SETDB1; however, the association of SETDB1 with the mutant p53 appears to be stronger than that with the wild type (Supplementary Fig. 5).

**Figure 5:**
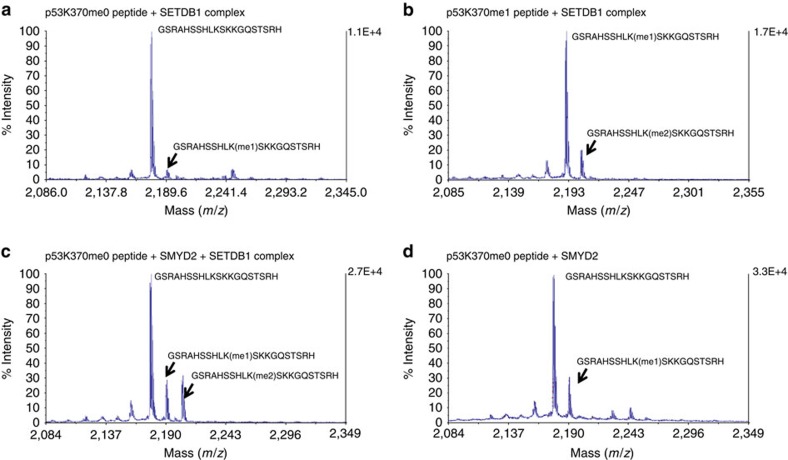
LC-MS/MS analysis of the p53 methylation by SETDB1. Unmethylated or chemically methylated p53 peptides were incubated with the endogenous SETDB1 complex. The end products were analysed using LC-MS/MS to confirm the p53 methylation. Corresponding peptide sequences and methylation status were marked by the identified peaks. (**a**) p53K370me0 peptide was used as the substrate. (**b**) K370me1 peptide was used as the substrate. (**c**) SMYD2 was used to induce K370me1. SETDB1 mainly catalyses p53K370me1 to K370me2. (**d**) SMYD2 alone with the p53K370me0 peptide was used as a control.

It has been shown that HMTs, such as KMT7, KMT3C, KMT5A and EHMT2, can methylate p53 at distinct sites^10^,^11^,^13^,^14^. Therefore, we decided to test whether SETDB1 could methylate p53. We first assessed p53 methylation by liquid choromatography with tandem mass spectrometry (LC-MS/MS) analysis on synthetic p53 peptides reacted with the SETDB1 complex. HCCLM3 cells were pretreated with Doxorubicin before SETDB1 pull down, and the methylase activity of the SETDB1 pull down was first confirmed with a histone methylation assay (Supplementary Fig. 6) We then examined *in vitro* p53 methylation by such endogenous SETDB1 complex using the p53 peptide as the substrate and S-adenosyl methionine as the methyl donor. We only detected a small peak of mono-methylation production from the unmethylated p53 substrate (Fig. 5a). Dot blot analysis indicated that this mono-methylation is p53K370me1 (Supplementary Fig. 7). Substantial production of K370me2 was observed using synthetic K370me1 peptide as the substrate (Fig. 5b and Supplementary Fig. 8). Consistently, peptides pretreated with SMYD2 (induces K370me1) also led to a significant production of K370me2 by the SETDB1 complex (Fig. 5c). As a control, SMYD2 alone monomethylated p53K370 as previously reported (Fig. 5d). These data suggest that SETDB1 could be a p53 methylase that mainly converts K370me1 to K370me2.

**Figure 6:**
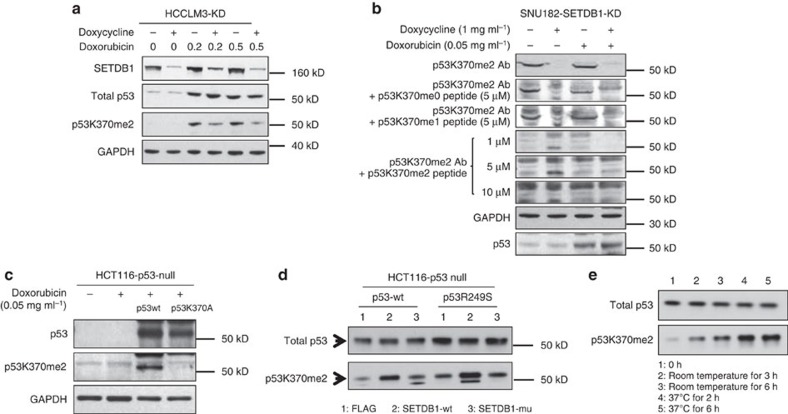
Regulation of p53K370 methylation by SETDB1. (**a**) To confirm that endogenous SETDB1 methylates endogenous p53, HCCLM3 cells stably infected with inducible SETDB1 shRNA were treated with doxycycline for 3 days, and then exposed to Doxorubicin for 24 h. Methylation of p53 was assessed using the antibody against p53K370me2. (**b**) SNU182 cells stably infected with inducible SETDB1 shRNA were treated with or without 1 μg ml^−1^ doxycycline for 3 days after transfection and then exposed to 0.05 μg ml^−1^ Doxorubicin for 24 h. The cells were harvested for western blot analysis using p53K370me2 antibody (1.2 μg ml^−1^) alone or competed with 5 μM p53K370 unmethylated or monomethylated peptides or di-methylated peptide at the dose of 1, 5 and 10 μM. All competing peptides were of 31-mer long in length. (**c**) p53 null HCT116 cells were transfected with wild-type p53 or a mutant p53 with K370 replaced with A (p53K370A). Cells were also treated with 0.05 μg ml^−1^ Doxorubicin for 24 h and harvested for western blot analysis using p53K370me2 antibody. GAPDH was analysed as the control. (**d**) p53 null HCT116 cells were transfected with wild type or p53R249S mutant together with SETDB1 or the set domain-deleted SETDB1 control. The methylation of p53 at K370 was measured by western blot analysis. (**e**) *In vitro* p53 methylation assays were performed using the endogenous SETDB1 complex pulled down from HCCLM3 cells that were treated with 0.5 μg ml^−1^ of Doxorubicin for 6 h. The immunoprecipitated complex was used as the enzyme in the assay with full-length p53 protein as the substrate and S-adenosyl methionine (SAM) as the methyl donor. Methylation was assayed using western blot analysis. The reaction was carried out at room temperature or at 37 °C for the duration as indicated.

We next tested methylation of endogenous p53 by endogenous SETDB1. Knockdown of SETDB1 reduced the K370me2 level in HCCLM3 cells (Fig. 6a). To validate the specificity of the p53K370me2 antibody, we performed peptide competition assay. The putative p53K370me2 bands were only competed away by the synthetic p53K370me2 peptides in a dose-dependent manner, but not by any other p53 peptides tested (Fig. 6b). By using a K370A-mutant p53, we observed no bands detected with the antibody (Fig. 6c), indicating that p53K370me2 was specifically recognized by the antibody. While endogenous SETDB1 can methylate either mutant or wild-type p53, mutant p53 appears to be methylated to a higher degree (Supplementary Fig. 5).

To address whether methylation of p53K370 is mediated by SETDB1 methyltransferase activity, we transfected HCT116 p53 null cells with p53 together with wild-type or catalytically dead SEDTB1. Overexpressed SETDB1 methylated either wild-type or mutated exogenous p53 at K370me2; however, the set domain-truncated SETDB1 had no effect on p53 methylation (Fig. 6d), indicating that the set domain of SETDB1 is required for p53K370 methylation. To ask whether SETDB1 can directly methylate full-length p53, we performed the *in vitro* methylation assay using the endogenous SETDB1 pull down complex and recombinant wild-type p53 protein. Indeed, the SETDB1 complex catalysed p53K370 to di-methylation (Fig. 6e).

### SETDB1 regulates p53 protein stability

**Figure 7:**
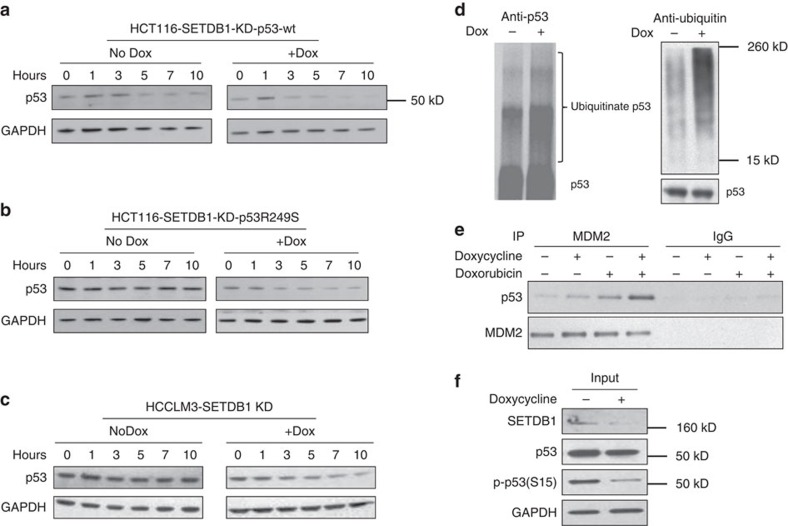
Regulation of p53 stability by p53K370 di-methylation. Wild-type p53 (**a**) or p53R249S (**b**) were transfected to p53 null HCT116 cells with stably infected inducible SETDB1 shRNA. The cells were treated with or without doxycycline for 3 days after transfection. Then, the cells were treated with 50 μg ml^−1^ cycloheximide and the turnover of p53 was measured at the time points indicated by western blot analysis using the antibody against total p53. (**c**) Similar experiments were performed to analyse the endogenous p53 turnover in HCCLM3 cells without p53 overexpression on SETDB1 knockdown. (**d**) SETDB1 knockdown increases p53 ubiquitination. HCCLM3 cells were treated with doxycycline as described above, and the cells were harvested for p53 immunoprecipitation and western blot analysis for p53 (left) or ubiquitin (right). The loading was normalized to total p53. (**e**) HCCLM3 cells infected with inducible SETDB1 shRNA were induced SETDB1 knockdown first and then treated with Doxorubicin before being harvested for immunoprecipitation. The cell lysates were immunoprecipitated with antibodies against total MDM2 or IgG control. The samples were then analysed using western blot analysis. Increased p53/MDM2 association was observed on SETDB1 knockdown. (**f**) SETDB1 knockdown reduces p53S15 phosphorylation. HCCLM3 cells were treated with doxycycline as described above, and the cells were harvested for western blot analysis on p53 phosphorylation (S15).

It has been shown previously that the methylation of p53 might affect p53 protein stability^10^. We thus asked whether di-methylation of p53 at K370 would be involved in the regulation of p53 stability. We introduced wild-type or mutated p53 to HCT116 p53 null cells, treated the cells with cycloheximide, a protein synthesis inhibitor, and measured the turnover of the p53 protein using western blot analysis. As expected, we observed a very rapid disappearance of the wild-type p53 (Fig. 7a) with the half-life estimated to be ∼3 h. In contrast, the R249S-mutant form of p53 was much more stable (Fig. 7b) with an estimated half-life of over 10 h. The turnover rate of p53R249S was accelerated by knocking down SETDB1 (Fig. 7b). Consistent evidence was seen in the HCCLM3 cells, where SETDB1 knockdown also promoted the degradation of the endogenous p53R249S (Fig. 7c).

We next examined whether this effect of SETDB1 knockdown on p53 stability went through MDM2-mediated ubiquitination. We found that SETDB1 knockdown increased p53 protein ubiquitination (Fig. 7d). Consistently, SETDB1 knockdown increased the association of MDM2 with p53 (Fig. 7e). Because the phosphorylation of p53 at S15 stabilizes p53 by preventing it from ubiquitination^28^, we next probed the level of p53 phosphorylation (S15) on SETDB1 knockdown. Inhibition of SETDB1 reduced the level of S15 phosphorylation of p53 (Fig. 7f). Together, these data suggest that SETDB1 regulates p53 stability presumably through the alteration of the K370 di-methylation status.

### Knockdown of SETDB1 inhibits HCCLM3 cell growth *in vivo*

**Figure 8:**
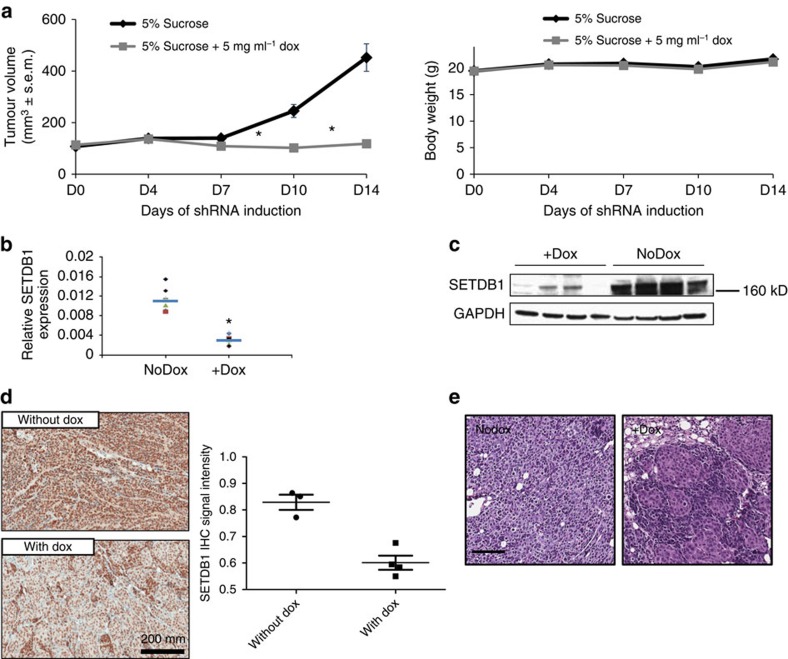
Inhibition of SETDB1 suppresses HCC cancer cell growth *in vivo*. (**a**) HCCLM3 was stably infected with lentiviral SETDB1 shRNA. Overall, 3 × 10^6^ microplasma-free cells were subcutaneously injected to the flank region of the female athymic nude mice. When tumour size reached 100 mm^3^, mice were randomized and fed with doxycycline-containing drinking water (5% sucrose with 0.5 mg ml^−1^ doxycycline, *n*=12) or sucrose only as the control (*n*=12). Doxycycline was changed twice every week. Tumour growth was measured using caliper. Body weight was monitored simultaneously. Data are presented as mean±s.e.m. *P*<0.001, *t*-test. (**b**) Total RNA was isolated from the xenograft tumour samples. Target knockdown in doxycycline-treated tumours was confirmed using RT–qPCR analysis as shown in the scatter plot. The blue line indicates the mean of each group. *P*<0.001, *t*-test, *n*=8. (**c**) Each of the four doxycycline-treated tumours or the control tumours were taken out for western blot analysis using antibody against SETDB1. Inhibition of SETDB1 protein expression was confirmed in doxycycline-treated tumours. (**d**) Immunohistochemical staining of SETDB1 in paraffin section of the xenograft tumour samples confirmed the target knockdown in doxycycline-treated tumours. Scale bar, 100 μM. The SETDB1 IHC signal was quantified using the Aperio algorithm. (**e**) Xenograft paraffin blocks were prepared and an H&E staining was applied to the sections for the pathological analysis. SETDB1 knocked down tumours appeared to be more differentiated than the controls. Shown here are representative fields. Scale bar, 100 μM.

We next assessed the tumour growth rate in HCCLM3 xenograft model in nude mice on SETDB1 knockdown. *In vivo* tumour growth was dramatically retarded by induction of SETDB1-inducible shRNA (Fig. 8a), without significant drop of body weight. SETDB1 knockdown efficiency was confirmed using RT–qPCR, western blot and IHC analyses using the tumour samples taken from the end of study (Fig. 8b–d).

Poorly differentiated tumours are often associated with poor prognosis. Since SETDB1 is important for mouse ES cell maintenance and differentiation^18^,^29^,^30^, we asked whether SETDB1 inhibition would induce tumour differentiation *in vivo*. We performed histopathology analysis of the tumours with or without SETDB1 knockdown (Fig. 8e). In the control group without doxycycline treatment (*n*=4), epithelial-like tumour cells were arranged in trabeculae and nests. These structures were manifested for ∼20–40% in three cases and very little, if any, in one case. In the doxycycline-treated, SETDB1 knocked down group (*n*=4), in addition to an increased abundance of trabeculae and nests, the tumour cells also formed a gland-like structure (Fig. 8e). These features took up more than 50% in three cases and ∼80% in one case. Together, our data suggest that SETDB1 inhibition leads to suppressed tumour growth and increased cell differentiation in HCCLM3 xenograft models.

## Discussion

**Figure 9:**
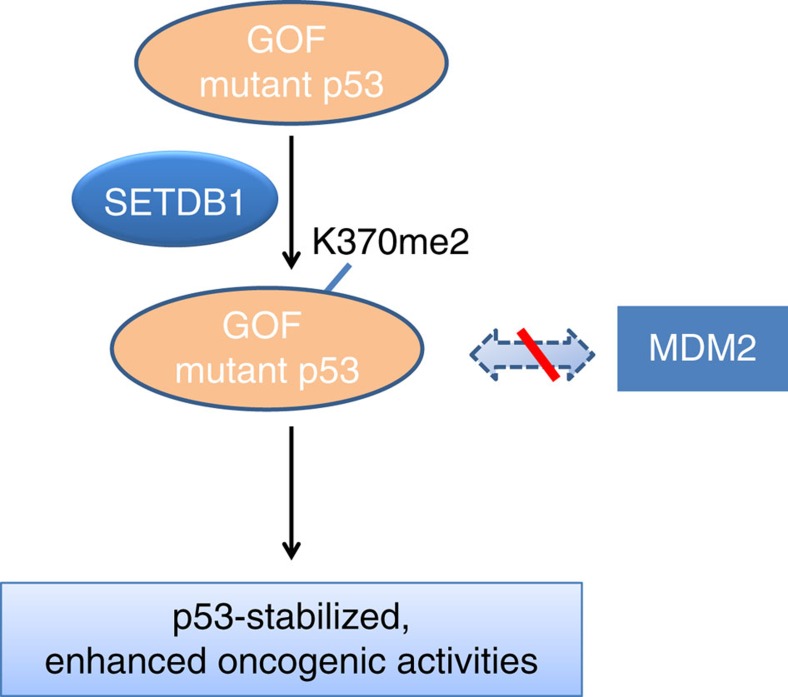
Working model of how SETDB1 regulates cancer cell growth through methylation of p53. GOF *TP53* mutations, such as R249S, are relatively stable and often oncogenic. The stability of these GOF p53 can be enhanced through interaction with and being di-methylated by SETDB1 at K370, which results in less MDM2 association. In cancer cells bearing GOF p53 mutation and *SETDB1* overexpression, attenuation of SETDB1 leads to less p53K370me2, enhanced p53 turnover and growth inhibition. Although SETDB1 can also interact with and methylate wild-type p53 at K370, it has little effect on cell growth as the wild type p53 is inherently very unstable and acts as a tumour suppressor. Therefore, SETDB1 can regulate cancer cell growth, at least in part, through methylation of GOF p53 at K370.

The present findings identified SETDB1 as a novel p53K370 di-methylase. SETDB1 di-methylates p53K370 and subsequently regulates p53 stability. While p53 stability can be regulated by a variety of modifications such as phosphorylation, acetylation, ubiquitination and methylation, the current findings suggest that SETDB1 is one of the important modulators involved in the p53 stability tuning. In cancer cells that harbour the GOF, oncogenic TP53 mutations, such as R249S, the p53 protein is stabilized by interaction with and being methylated by SETDB1. Stabilized oncogenic p53 by SETDB1 confers the cell growth advantages. On the other hand, wild-type p53 is inherently unstable. It is less associated with SETDB1 and less methylated. Since wild-type p53 is only present in cells at a very low level, methylation of the wild-type tumour-suppressive p53 by SETDB1 may contribute very little to cell growth. The current working model is depicted in a schematic drawing in Fig. 9.

The exact reason why a single point mutation of p53 such as R249S makes a difference of the level of association of p53 with SETDB1 is not known yet. We hypothesize that this can be explained in the following aspects: (1) wild type and mutant p53 may have distinct protein configurations and exist in different contexts (such as DNA-binding and so on) in cells. Therefore, the context where mutant p53 resides may favour the interaction with SETDB1. (2) While SETDB1 methylates both wild-type and mutant p53, mutant p53 may have more chances to get K370 methylation *in vivo* because it associates more tightly with SETDB1 in cells as we have seen (Supplementary Fig. 5). Therefore, the effect on mutant p53 stabilization by SETDB1 is more obvious than that on wild-type p53. (3) p53 stability is broadly regulated by a variety of other modifications such as phosphorylation and acetylation. The mutant p53 is constitutively more stable, while the wild-type p53 only exists in cells at a low level. In our study, SETDB1 may function as a modulator to tune p53 degradation. As the binding of SETDB1 to mutant or wild-type p53 differs in cells, the consequent different status on K370 methylation exaggerates the stability difference between mutant and wild-type p53. As a result, mutant p53 seems to be more dependent on SETDB1.

Our working model also predicts that cancer cells with other GOF *TP53* mutations than R249S might also gain growth advantage in the presence of high *SETDB1* expression. This was shown in the SNU182 cells with mutant *TP53* (S215I+P72R) and high *SETDB1* expression. Thus, we propose that SETDB1 regulates cancer cell growth by methylating GOF-mutant p53.

It is known that p53 can be mono-methylated by SMYD2. However, p53K370me1 does not affect protein stability but abolishes its wild-type activity^11^,^12^. In this study, we showed that SETDB1 could affect p53 stability through K370 di-methylation, suggesting that p53K370me2 has distinct roles from p53K370me1. Preliminary evidence suggested that SETDB1 might also methylate p53 at K372 and regulate its stability when K370 is mutated and is under overexpression conditions. One possibility is that K370 is the primary methylation site and K370me2 may block K372 methylation by SETDB1. Therefore, K372 methylation can only happen when K370 is not methylated. The selectivity and substrate specificity of SETDB1 on p53 methylation as well as potential context-dependent nearby residue interaction warrant future investigation. Besides methylation, p53 could also be modified by acetylation, phosphorylation and so on. It has been shown that different forms of post-translational modifications of p53 interact with each other. For example, KMT7-mediated p53K372 methylation prevents p53K370 methylation mediated by SMYD2 (ref. 12) but facilitates p53K373/K382 acetylation^31^. How K370 di-methylation may interact with other p53 modifications remains elusive and warrants future study.

Although SETDB1 mediates histone H3K9 (ref. 16) tri-methylation as a histone methyltransferase, it remains to be determined whether SETDB1 can also tri-methylate p53 at K370. Tri-methylation was not observed in our *in vitro* methylation assay; however, it could be due to insufficient SETDB1 enzymatic activity or lack of cofactors in the *in vitro* environment.

That p53 can be methylated and demethylated by HMTs and HDMs, respectively, not only reveals new roles of HMTs and HDMs other than histone methylation but also demonstrates the molecular similarity between histone modification and their non-histone substrate methylation^10^,^32^. Many non-histone protein substrates have been identified for various HMTs such as EHMT2 (ref. 33). However, very little is known about non-histone substrates of SETDB1. In the current study, we identified p53 as the first non-histone substrate of SETDB1, to the best of our knowledge. It would be interesting to explore additional non-histone substrates of SETDB1 to better understand its biological functions.

SETDB1 is responsible for the repressive H3K9me3 modification^29^,^30^,^34^ and regulates the expression of tumour suppressors *P53BP2* and *RASSF1A*. *SETDB1* is located at chromosome 1q21, which is recently identified as a melanoma susceptibility locus^22^. In a screen in zebrafish melanoma models, SETDB1 was found to contribute to tumorigenesis in a p53 null background with activated rat sarcoma virus oncogene (RAS)^23^. However, the underlying molecular mechanism is not fully understood. Our study provides a novel mechanism by which SETDB1 regulates cancer cell growth through the modulation of p53 methylation. These results, however, do not exclude the possibility that the transcription regulation by SETDB1 via histone H3K9 methylation may also contribute to cancer cell growth control. Recently, it was also reported that p53 suppresses SETDB1 gene expression during paclitaxel-induced cell death^35^. Comprehensive epigenomic and transcriptional profiling to understand how SETDB1 regulates gene expression in cancer cells warrants future study.

Aflatoxin, a known mycotoxin, is one of the strongest carcinogens for liver cancer^36^,^37^. The risk for Hepatitis B virus carriers with aflatoxin exposure to develop liver cancer compared with unexposed individuals is much greater than aflatoxin or hepatitis B virus alone^38^. It is known that Aflatoxin B could cause *TP53* mutations, including the hotspot R249S mutation^4^,^39^. Our data are consistent with previous reports that R249 is highly mutated in Chinese liver cancer patients. The high-frequency missense mutations of *TP53* in cancers support a critical and complex role of *TP53* in tumorigenesis. The GOF *TP53* mutations are functionally independent of wild-type p53, and the accumulation of mutated p53 contributes to tumorigenesis^1^,^6^. Therefore, clearance of mutated p53 is of critical importance for prevention or treatment of cancer. Using exome sequencing and genomic profiling, we found that moderate *SETDB1* copy number gain is associated with enrichment of *TP53* mutations, with the GOF R249S being the most frequent. These data suggest that tumour cells with SETDB1 copy number gain/overexpression along with R249S *TP53* mutation may acquire growth advantages through genetic mechanisms. This hypothesis is consistent with our findings that R249S mutant, but not wild-type p53, confers cell sensitivity to SEDTB1 attenuation. Together, our data revealed a novel function of SETDB1 to regulate HCC cell growth through p53K370 di-methylation.

## Methods

### Cell culture and proliferation assay

Human cancer cell lines HCCLM3, SNU182, Hep3B and HCT116 were obtained from ATCC and were cultured as instructed. Hep3B and SNU182 cells were maintained in DMEM/F12 medium. HCCLM3 cells were maintained in DMEM medium. HCT116 cells were maintained in MyCoy′5a medium. All the media were supplemented with 10% fetal bovine serum and 1% penicillin and streptomycin (all from Invitrogen). Cells were maintained under a standard gas atmosphere of humidified air/5% CO_2_. All the cell lines were tested for mycoplasma contamination and authenticated with PCR genotyping using GenePrint STR System (Promega, DC6451).

CellTiter-Glo (Promega) was used for cell growth measurement. Cells were seeded in 96-well plate in 100 μl medium for each well. For inducible SETDB1 knockdown, 1 μg ml^−1^ doxycycline was added in the medium. After cells were cultured for a given period, 100 μl per well CellTiter-Glo reagent was added to measure cell growth according to the manufacturer's instruction.

### RNA interference

Two independent siRNAs were used to knock down SETDB1. The respective sequences are si-Q3: 5′- CAGCATGCGAATTCTGGGCAA -3′, si-Q4: 5′- TCGGGTGGTCGCCAAATACAA -3′, Ctrl-siRNA: siGLO RISC-Free Control siRNA (Dharmacon). siRNAs were transfected using Lipofectamine RNAiMAX (Invitrogen) and assayed for 3–5 days after transfection. The shRNA against p53 was ordered from Sigma (Clone ID: NM_000546.4-887s21c1). The sequence is: 5′- CCGGCACCATCCACTACAACTACATCTCGAGATGTAGTTGTAGTGGATGGTGTTTTTG -3′.

For inducible shRNA, the shRNA sequence was cloned into the single lentiviral-based Tet-on-inducible vector pLKO-Tet-on system (Sigma) as previously reported^40^. The sequences of shRNAs that target SETDB1 are: sh1-5′: 5′- CCGGGTTCGGCTACAGCTATTCACTCGAGTGAATAGCTGTAGCCGAACTTTTT -3′; sh1-3′: 5′- AATTAAAAAGTTCGGCTACAGCTATTCACTCGAGTGAATAGCTGTAGCCGAAC -3′; sh2-5′: 5′- CCGGGATGTGAGTGGATCTATCGCTCGAGCGATAGATCCACTCACATCTTTTT -3′; sh2-3′: 5′- AATTAAAAAGATGTGAGTGGATCTATCGCTCGAGCGATAGATCCACTCACATC -3′. Each pair of shRNAs was annealed and inserted into the pLKO-Tet-on vector and co-transfected into 293T with pCG10 and pCG41. Cell supernatants were collected 48 h after transfection and passed through a 0.22-μm filter. Cells were infected with the virus supernatant and were cultured in medium containing 2 μg ml^−1^ puromycin for stable cell line selection.

### Immunoprecipitation

Immunoprecipitation after formaldehyde crosslinking was performed as previously described^41^ with minor modifications. Briefly, cells were treated with 1% formaldehyde for crosslinking for 10 min at room temperature. Cells were harvested and lysed in 1 × RIPA buffer. The samples were sonicated until the lysate became clear, followed by centrifugation at 15,000*g* for 15 min at 4 °C. The supernatant was collected for immunoprecipitation (IP) using antibodies against SETDB1 (H-300; Santa Cruz, 1:250) and p53 (7F5; Cell Signaling, 1:500). The magnetic protein G beads after IP were washed three times with 1 × RIPA buffer and proteins were eluted with 2 × SDS-loading buffer. The samples were then incubated at 99 °C for 20 min before sample loading for SDS–PAGE. For non-crosslinking p53 immunoprecipitation, it was performed similarly except for the crosslinking procedure. Cells were transient transfected with wild-type p53 or p53R249S mutants. Forty-eight hours after transfection, cells were harvested and processed for IP using the Direct IP Kit (Pierce). For endogenous SETDB1 complex isolation with IP, the Nuclear Complex Co-IP Kit (Active Motif) was used for the procedure, with anti-SETDB1 antibody (Santa Cruz, 1:250) being added to the IP reaction.

### Western blot analysis

Western blot analysis was performed as described previously^42^. Samples were collected directly in 1 × NuPAGE LDS sample buffer with 1 × Sample reducing buffer (Invitrogen) and denatured at 95 °C for 5 min followed by a centrifugation at 18,500*g* for 5 min. The supernatant was electrophoresed on a 4–12% Tris-HCl gel and transferred to nitrocellulose membrane (Invitrogen). After blocking with Superblock T20 blocking buffer (Thermo Scientific), the membrane was incubated with a primary antibody overnight at 4 °C and then with a secondary antibody conjugated with alkaline phosphatase (1 h at room temperature); the signal was detected by using a chemiluminescence method. The following primary antibodies were used: anti-SETDB1 (Santa Cruz, 1:1,000), anti-p53 (Cell Signaling, 1:1,000), anti-p53/K370me1 and anti-p53/K370me2 (in-house made as previously described^32^; 1:1,000), anti-p53/K372me1 (ab16033, AbCAM, 1:1,000), anti-GAPDH (7B; Santa Cruz, 1:10,000), anti-H3K9me3 (39161, Active Motif, 1:1,000), anti-p-p53(S15; 16G8; Cell Signaling, 1:1,000) and anti-MDM2 (1F2; Millipore, 1:200).

### RNA isolation and real-time RT–PCR

RNA was extracted using the RNeasy Mini Kit (Qiagen). RNA quality was confirmed with Nanodrop. Real-time RT–PCR analysis was performed on an ABI Prism 7900 Sequence Detection System using the SYBR Green PCR Master Mix (Applied Biosystems, Foster City, USA). The relative expression of each gene was normalized against GAPDH. The primers used for the quantitative RT–PCR are shown in Supplementary Table 1.

### TMA and immunohistochemistry

For human HCC, TMAs were constructed from 59 Chinese HCC cases. Out of the 59 cases, 35 have both tumour and adjacent samples (2 cm away from tumour structure) and 4 of the 35 cases have both adjacent and distal samples (5 cm away from tumour structure). Human samples were obtained with patient-informed consent under protocols approved by the Changhai Hospital. Formalin-fixed, paraffin-embedded tissue sections (4-μm thick) are prepared with tumour and adjacent/distal samples from a given case are placed side by side on the TMA. Haematoxylin and eosin staining of the TMA section was conducted and reviewed to confirm that the presence of tumour cells is more than 80% and that no tumour structures included in the adjacent/distal samples. TMA was subjected to immunohistochemistry by using Ventana Discovery-automated slide stainer (Ventana Medical Systems) with SETDB1 antibody (4A3; Sigma, 1:1,000). For EdU immunofluorescence, the labelling and detection were performed using the Click-iT EdU Imaging kit according to the manufacturer's instruction (Invitrogen).

### TP53 sequencing and mutagenesis

Total RNA was extracted from HCCLM3 cell pellets. cDNA was made using Superscript III (Invitrogen, Cat. No. 18080-051) and used as the template for p53 PCR. The sequences of the primers are listed in Supplementary Table 1. The PCR product was purified using the PCR purification Kit (Qiagen, Cat. No. 28106) and sequenced by Invitrogen. The sequence analysis covers the whole p53 open reading frame. For mutant p53 construction, p53 full-length coding region was isolated from HCCLM3 by PCR with 5′-BamHI and 3′-XhoI restriction enzyme sites added to the flank. The mutant and wild-type p53 was placed into pcDNA3.1(+) with Myc and His tags. The recombinant plasmids were confirmed with DNA sequencing.

PCR-based mutagenesis was applied to generate p53K370 mutation. Wild type or p53R249S constructs in pcDNA3.1 were used as the template for PCR using Phusion HF 2* master mix (NEB), with specific primers to introduce the K370A mutation. After 15 cycles of PCR, 1 μl DpnI (NEB) was added into the PCR reaction and further incubated at 37C for 1 h. The reaction mixture was transformed top10 competent cells (Invitrogen). Single colonies were selected and the mutant sequence was verified with DNA sequencing.

### Fragment length polymorphism genotyping of human HCC samples

Formalin-fixed paraflin embedded (FFPE) blocks were collected from a local hospital (Changhai Hospital). Human samples were obtained with patient-provided informed consent under protocols approved by the Changhai Hospital. The QIAamp DNA FFPE Tissue Kit (Qiagen) was used for DNA extraction from the tissues. The fragments around codons 249 and 72 were amplified with PCR using the Platinum PCR SuperMix High Fidelity (Invitrogen). For condon 72, nest PCR was adapted to increase specificity using p53 9 and 12 primers. The condon 249 and codon 72 PCR products were digested with HaeIII and BstUI, respectively, and analysed using electrophoresis in 10% TBE polyacrylamide gel. The primers are listed in Supplementary Table 1.

### *In vitro* methylation assay and LC-MS/MS analysis

The *in vitro* methylation assay was similar to previously described literature^10^,^32^. Briefly, full-length p53 protein (100 nM) or peptides (10 nM) were incubated with recombinant SETDB1 protein (400 nM) or endogenous SETDB1 complex at room temperature or 37 °C. For the assay using the full-length p53 protein, the reaction products were separated on SDS–PAGE and detected with western blot analysis using antibody against p53 (Cell Signaling), or p53K370me1 or p53K370me2 (in-house made).

For the LC-MS/MS analysis, chemically modified p53K370me1 or p53K370me0 peptides (10 nM) were incubated with the endogenous SETDB1 complex at 37 °C for 6 h in a reaction buffer containing 20 mM Tris-HCl, 5 mM MgCl_2_, 100 mM NaCl, 0.1% Tween-20, 1 mM dithiothreitol and 10 μM S-adenosyl methionine. The p53 peptides with and without K370 methylation were synthesized from GL Biochem Ltd (Shanghai, China). The end products were subsequently detected by dot blot or LC-MS/MS analyses. The MS experiment was performed on LTQ VELOS (Thermo Finnigan, San Jose, CA) with positive electrospray ionisation (ESI) using 0.15 mm × 150 mm (RP-C18) column (Column Technology Inc.) and Zorbax 300SB-C18 peptide trap column (Agilent Technologies, Wilmington, DE). The mobile phase was 84% acetonitrile in 0.1% formic acid with a gradient elution of acetonitrile from 4 to 60% (0–20 min), 60 to 100% (20–30 min) and 100% (30–40 min). MS data were acquired by full-scan (M/Z 600–1,200) followed by 20 MS2 scan. In addition, the raw file data were analysed with BIOWORKS.

### Bioinformatics and genetic analysis

Clinical cancer gene expression profiling data were downloaded from gene expression omnibus (GEO) or European Bioinformatics Institute (EBI) including malignant pleural mesothelioma (GSE2549), renal cell cancer (GSE15641), renal tumours (GSE11024), urinary bladder carcinoma (GSE3167), breast cancer (GSE5847), HCV-infected HCC (GSE6764), glioma (GSE4290), kidney cancer (GSE781) and prostate cancer (E-TABM-26). The expression signals of SETDB1 were extracted and analysed with box-plots using Prism. For samples profiled using the Affymetrix platform, the probe set ID (203155_at) was used to check SETDB1 expression.

For DNA copy number analysis, the GISTIC algorithm^24^ was used to calculate a G-score for each amplification or deletion region based on its frequency and amplitude. The statistical significance (*q* value) was computed to indicate the likelihood of these regions to represent a driver aberration, as opposed to a random aberration.

The *TP53* mutation status was extracted from the Asian gastric primary tumour xenograft models and fresh Asian HCC primary tumour tissue samples by using exome sequencing. Genomic DNA was extracted from frozen tumour tissues using the Qiagen DNeasy kit (Cat. No. 69504). Gene exons were then captured with Agilent SureSlect (50 Mb) reagents and were further subjected to 100 × coverage of deep sequencing on Illumina MiSeq platform following the manufacturer's instruction. Whole-exome sequencing data were aligned and mapped to the human genome using Burrows–Wheeler Aligner. Single-nucleotide variants and small insertion/deletions were called using GATK 1.0.

### *In vivo* xenograft study

The *in vivo* animal procedure was approved by the Animal Ethics Committee at the Novartis Institutes for Biomedical Research. HCCLM3 stably infected with the inducible shRNA against SETDB1 was grown to the log phase. 3 × 10^6^ mycoplasma-free cells were subcutaneously injected to the flank region of the 6-week female athymic nude mice. When tumour size reaches 100 mm^3^, mice were randomized and fed with doxycycline-containing drinking water (5% sucrose with 0.5 mg ml^−1^ doxycycline, *n*=12) or sucrose only as the control (*n*=12). Doxycycline was changed twice every week. Tumour growth was measured using caliper. Body weight was monitored simultaneously.

## Additional information

**How to cite this article:** Fei, Q. *et al*. Histone methyltransferase SETDB1 regulates liver cancer cell growth through methylation of p53. *Nat. Commun.* 6:8651 doi: 10.1038/ncomms9651 (2015).

## Supplementary Material

Supplementary Information Supplementary Figures 1-8 and Supplementary Table 1

## Figures and Tables

**Table 1:** Association of TP53 mutation with SETDB1 amplification in liver cancers.

	**SETDB1 over-expression/amplification** **TP53 mut**	**SETDB1 over-expression/amplification TP53 wt**	**SETDB1 normal TP53 mut**	**SETDB1 normal TP53 wt**	*P **value**
Fresh liver cancer	12	6	25	41	0.03

Mut, mutated; wt, wild type.

^*^Fisher's exact test
