# Supplementary material for: Histone methyltransferase SETDB1 regulates liver cancer cell growth through methylation of p53
Source: Nat Commun. 2015 Oct 16;6:8651. doi: 10.1038/ncomms9651 (PMC5426523; doi:10.1038/ncomms9651)
Supplement: Supplementary Information — Supplementary Figures 1-8 and Supplementary Table 1 [file ncomms9651-s1.pdf]

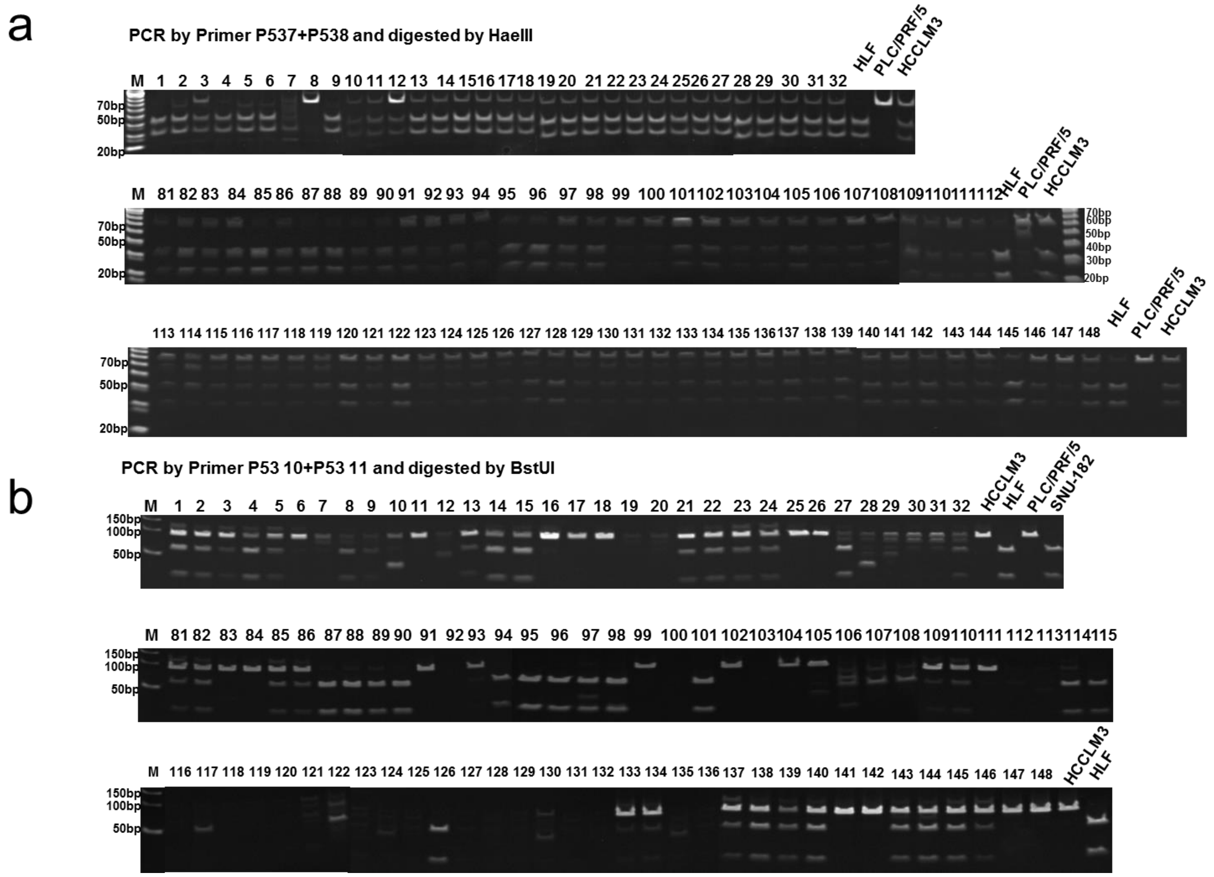

Supplementary Fig. 1│ Genotyping of *TP53* in Chinese primary HCC tumor samples by RFLP analysis. Genomic DNA was extracted from FFPE sections of HCC patient samples collected from local hospital using QIAamp DNA FFPE Tissue kit and used as the template for PCR for codon 249 (**a**) or 72 (**b**). The codon 249 and 72 PCR products were digested by HaeIII and BstUI, respectively and analyzed by electrophoresis in 10% TBE polyacrylamide gel.

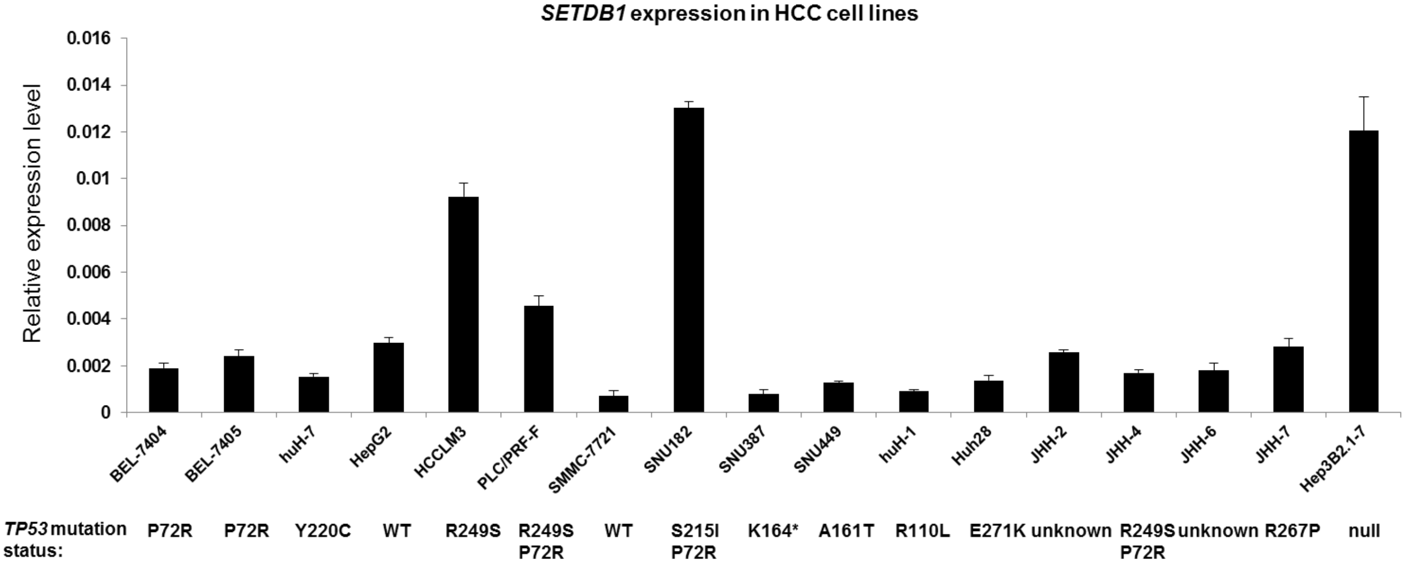

Supplementary Fig. 2 │Profiling of *SETDB1* expression in human HCC cancer lines. Total RNA was isolated from a panel of HCC cell lines. The expression of SETDB1 in these samples was analyzed by RT-qPCR. The relative expression level of SETDB1 was normalized to GAPDH. Data are presented as mean ± s.d. (n=3). The *TP53* mutation status was also marked.

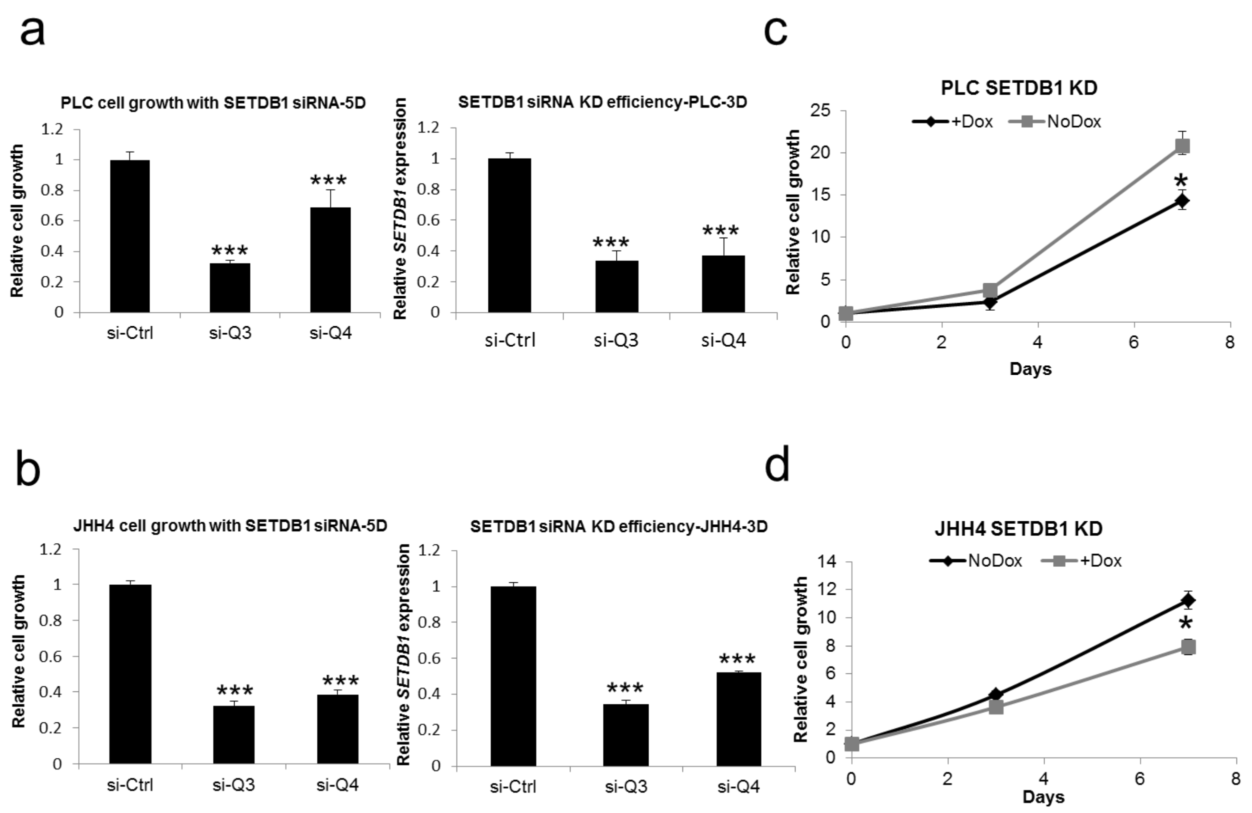

Supplementary Fig. 3 │SETDB1 Knockdown inhibits PLC/PRF and JHH4 cell proliferation. R249S bearing PLC/PRF and JHH4 cells were either treated with SETDB1 siRNAs or inducible shRNA. The growth was examined by CellTiter-Glo (n=6). The knockdown efficiency of siRNA or shRNA was confirmed by RT-qPCR (n=3). Data are presented as mean ± s.d. *, $p < 0.001$, t test.

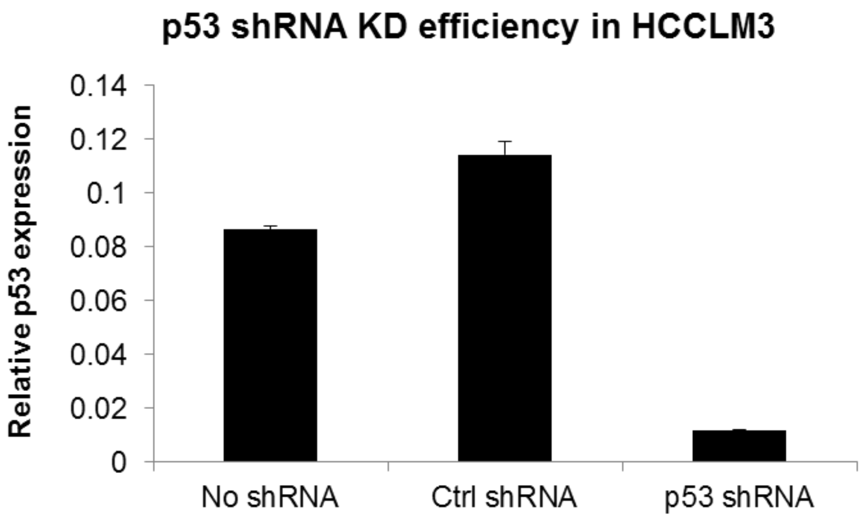

Supplementary Fig. 4│Knockdown efficiency of p53 shRNA. HCCLM3 cells were transfected with shRNA against*TP53*. 3 days after transfection, the cells were harvested for RT-qPCR analysis of *TP53* expression. The relative expression level of *TP53* was normalized to GAPDH. Data are presented as mean ± s.d. (n=3).

a

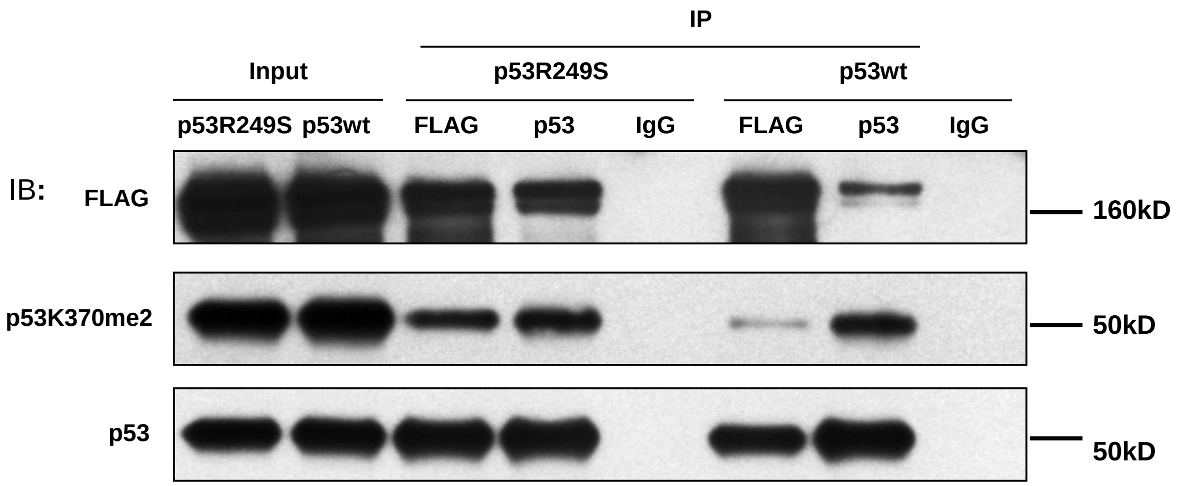

b

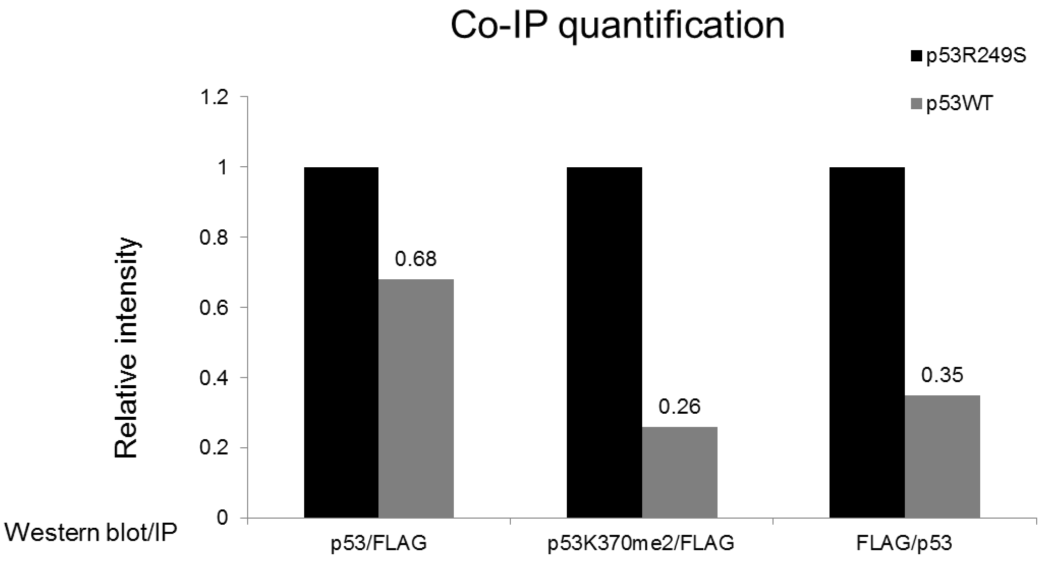

Supplementary Fig. 5│ SETDB1 complexes with p53 in cells. HCT116 p53 null cells were transfected with Flag-tagged SETDB1 together with wild type or R249S mutated p53. Cells were collected for immunoprecipitation using antibodies against FLAG for SETDB1 detection or total p53. **a,** the samples were then analyzed using Western blot analysis. The association of mutant p53 with SETDB1 appears to be stronger than that of wild type p53 and the mutant p53 is also methylated to a higher degree. **b,** densitometry measurement of the relatively intensity of the bands.

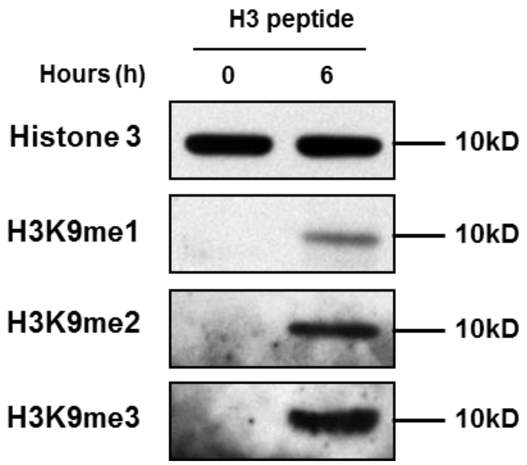

Supplementary Fig. 6│ Confirmation of methylase activity of the endogenous SETDB1 complex. HCCLM3 cells that were treated with 0.5 µg/ml of Doxorubicin for 6 hours prior to SETDB1 pull down preparation. The SETDB1 immunoprecipitated complex was used as the enzyme in the *in vitro* methylation assay with synthetic H3 peptide (1-84aa) as the substrate and SAM as the methyl donor. Methylation was assayed using Western blot analysis. The reaction was carried out at 37 ^{o}C for 6 hours. The SETDB1 pull down complex methylates histone H3K9 to mo, di, and tri-methylation.

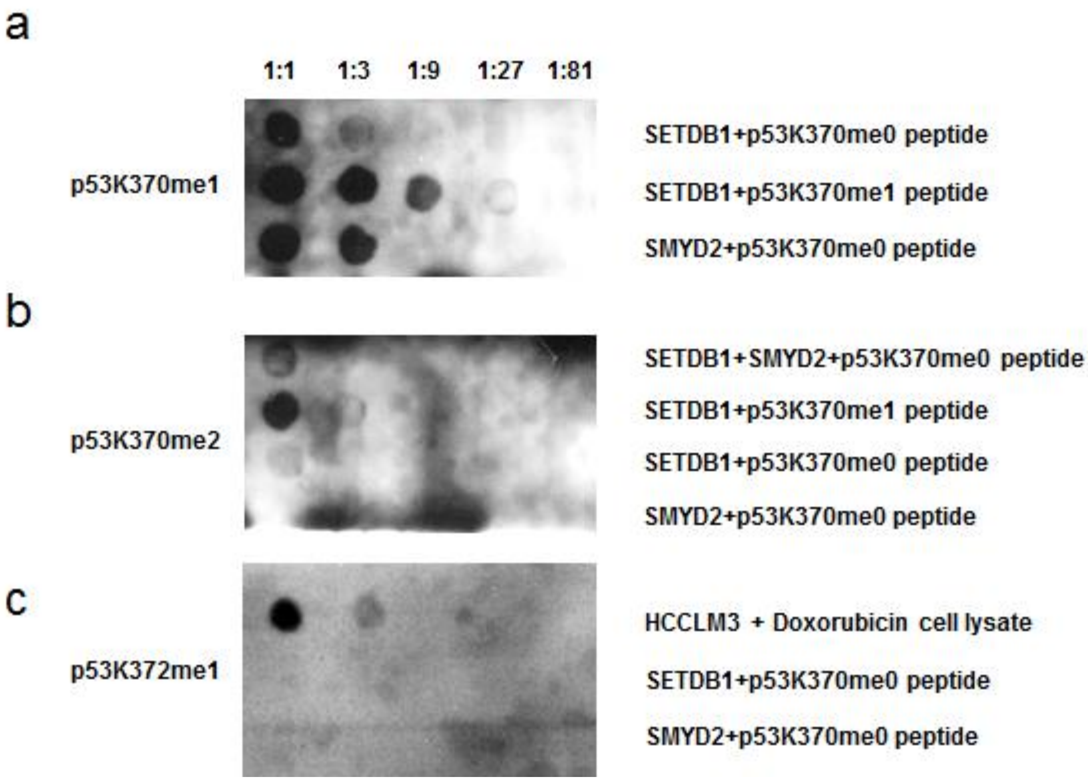

Supplementary Fig. 7│ Dot blot confirmation of p53 methylation by SETDB1 complex. The *in vitro* methylation assays were performed as described in the method section. The end products of the peptide based assay were further analyzed by dot blot analysis using the antibody against p53K370me1 (**a**), p53K370me2 (**b**), or p53K372me1 (**c**). We used whole cell lysate from HCCLM3 treated with doxorubicin as the positive control. The results indicate that SETDB1 mainly methylates p53K370me1 to p53K370me2.

p53K370me1 peptide + SETDB1 complex

a

DTA: S10.2029.2029.3
Precursor ion: 730.31
Mass type: Average
Mod's: (M* +15.99940) (K# +14.01560) (K@ +28.03120)

Ion series for charge: +1

AA	A ions	B ions	B* ions	Bo ions	C ions	Y ions
G		58.06				
S		145.14				2134.34
R		301.32				2047.26
A		372.40				1891.07
H		509.54				1820.00
S		596.62				1682.86
S		683.70				1595.78
H		820.84				1508.70
L		933.99				1371.56
K#		1076.18				1258.40
S		1163.26				1116.21
K		1291.43				1029.14
K		1419.61				900.96
G		1476.66				772.79
Q		1604.79				715.74
S		1691.87				587.61
T		1792.97				500.53
S		1880.05				399.43
R		2036.23				312.35
H						156.16

c

DTA: S10.3110.3110.3
Precursor ion: 735.87
Mass type: Average
Mod's: (M* +15.99940) (K# +14.01560) (K@ +28.03120)

Ion series for charge: +1

AA	A ions	B ions	B* ions	Bo ions	C ions	Y ions
G		58.06				
S		145.14				2148.35
R		301.32				2061.28
A		372.40				1905.09
H		509.54				1834.01
S		596.62				1696.87
S		683.70				1609.79
H		820.84				1522.72
L		933.99				1385.58
K@		1090.20				1272.42
S		1177.28				1116.21
K		1305.45				1029.14
K		1433.62				900.96
G		1490.67				772.79
Q		1618.80				715.74
S		1705.88				587.61
T		1806.99				500.53
S		1894.06				399.43
R		2050.25				312.35
H						156.16

b

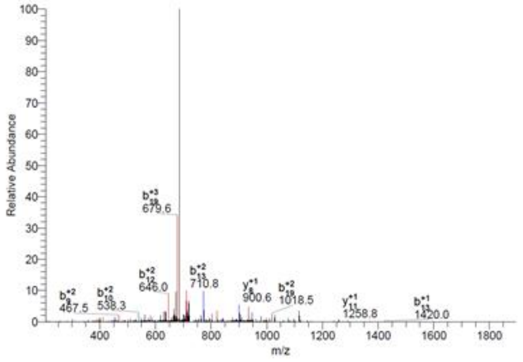

d

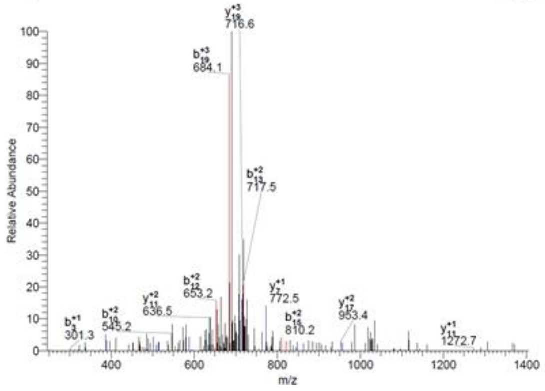

Supplementary Fig. 8│ LC-MS/MS analysis of *in vitro* methylation of p53 by SETDB1 complex. The methylation of p53 by SETDB1 was confirmed by LC-MS/MS analysis. Shown here is one representative data set of methylation of p53K370me1 substrate by SETDB1. **a,** Matched fragment ion for GSRAHSSHLK(me1)SKKGQSTSRH. **b,** MS/MS spectrum of GSRAHSSHLK(me1)SKKGQSTSRH. K# indicates K370me1. **c,** Matched fragment ion for GSRAHSSHLK(me2)SKKGQSTSRH. **d,** MS/MS spectrum of GSRAHSSHLK(me2)SKKGQSTSRH. K@ indicates K370me2.

Supplementary Table 1. Sequence of the primers used in the study

**Primers for RFLP genotyping of p53**	
P53 7	5'-AGTTCCTGCATGGGCGGCAT-3'
P53 8	5'-CTGACCTGGAGTCTTCCAGT-3'
P53 9	5'-CACTGAAGACCCAGGTCCAGA-3'
P53 10	5'-GAAGCTCCCAGAATGCCAGA-3'
P53 11	5'-CCAGGAGGGGGCTGGTGCAG-3'
P53 12	5'-GGAAGGGACAGAAGATGACAGG-3'
**Primers for RT-qPCR**	
SETDB1	5'- GGGCAAGGGTGTTTTCATTAAC -3'
	5'- GTTAGTTGATGGCAGGCACACTT -3'
p53	5'- GTTCCGAGAGCTGAATGAGG -3'
	5'- TCTGAGTCAGGCCCTTCTGT -3'
GAPDH	5'- CGACCACTTTGTCAAGCTCA -3'
	5'- AGGGGTCTACATGGCAACTG -3'
**Primers for cloning of p53 and mutagenesis**	
hP53-out-F	5'-CCACCGTCCAGGGAGCAGGTAGC-3'
hP53-out-R	5'-CTTCTGACGCACACCTATTGCAAG-3'
hP53-1F(BamHI)	5'-CGGGATCCATGGAGGAGCCGCAGTCAGATCC-3'
hP53-393R(XhoI)	5'-CCGCTCGAGGTCTGAGTCAGGCCCTTCTGTCT-3'
P53-K370A-F	5'-CACTCCAGCCACCTGGCGTCCAAAAAGGGTCAG-3'
P53-K370A-R	5'-CTGACCCTTTTTGGACGCCAGGTGGCTGGAGTG-3'
P53-K370A-F	5'-CCAGCCACCTGAAGTCCGCAAAGGGTCAGTCTACC-3'
P53-K370A-R	5'-GGTAGACTGACCCTTTGCGGACTTCAGGTGGCTGG-3'
